# TGF-*β* is an inducer of ZEB1-dependent mesenchymal transdifferentiation in glioblastoma that is associated with tumor invasion

**DOI:** 10.1038/cddis.2014.395

**Published:** 2014-10-02

**Authors:** J V Joseph, S Conroy, T Tomar, E Eggens-Meijer, K Bhat, S Copray, A M E Walenkamp, E Boddeke, V Balasubramanyian, M Wagemakers, W F A den Dunnen, F A E Kruyt

**Affiliations:** 1 Department of Medical Oncology, University of Groningen, University Medical Center Groningen, Groningen, The Netherlands; 2 Department of Pathology, University of Groningen, University Medical Center Groningen, Groningen, The Netherlands; 3 Department of Gynecologic Oncology, University of Groningen, University Medical Center Groningen, Groningen, The Netherlands; 4 Department of Neuroscience, University of Groningen, University Medical Center Groningen, Groningen, The Netherlands; 5 Department of Pathology, The University of Texas MD Anderson Cancer Center, Houston, TX 77030, USA; 6 Department of Neuro-surgery, University of Groningen, University Medical Center Groningen, Hanzeplein 1, 9713 Groningen, The Netherlands

## Abstract

Different molecular subtypes of glioblastoma (GBM) have been recently identified, of which the mesenchymal subtype is associated with worst prognoses. Here, we report that transforming growth factor-*β* (TGF-*β*) is able to induce a mesenchymal phenotype in GBM that involves activation of SMAD2 and ZEB1, a known transcriptional inducer of mesenchymal transition in epithelial cancers. TGF-*β* exposure of established and newly generated GBM cell lines was associated with morphological changes, enhanced mesenchymal marker expression, migration and invasion *in vitro* and in an orthotopic mouse model. TGF-*β*-induced mesenchymal differentiation and invasive behavior was prevented by chemical inhibition of TGF-*β* signaling as well as small interfering RNA (siRNA)-dependent silencing of ZEB1. Furthermore, TGF-*β-*responding and -nonresponding GBM neurospheres were identified *in vitro*. Interestingly, nonresponding cells displayed already high levels of pSMAD2 and ZEB1 that could not be suppressed by inhibition of TGF-*β* signaling, suggesting the involvement of yet unknown mechanisms. These different GBM neurospheres formed invasive tumors in mice as well as revealed mesenchymal marker expression in immunohistochemical analyses. Moreover, we also detected distinct zones with overlapping pSMAD2, elevated ZEB1 and mesenchymal marker expression in GBM patient material, suggestive of the induction of local, microenvironment-dependent mesenchymal differentiation. Overall, our findings indicate that GBM cells can acquire mesenchymal features associated with enhanced invasive potential following stimulation by secretory cytokines, such as TGF-*β*. This property of GBM contributes to heterogeneity in this tumor type and may blur the boundaries between the proposed transcriptional subtypes. Targeting TGF-*β* or downstream targets like ZEB1 might be of potential benefit in reducing the invasive phenotype of GBM in a subpopulation of patients.

Glioma is the most frequent primary tumor of the brain and is generally classified into four grades based on histology.^1^ Grade 4 gliomas, glioblastoma (GBM), are highly malignant, often associated with strong microvascular proliferation and necrosis and display strong infiltrating properties. Current standard treatment consists of surgery combined with radiotherapy and chemotherapy.^2^ However, the inability to resect all tumor cells together with resistance to therapy, including novel targeted agents, results in inevitable recurrent disease leading to a poor median survival of patients of 12–15 months.^3,4^

Recent lines of research have emphasized on a comprehensive genomic and epigenomic classification in GBM that should lay the groundwork for an improved molecular understanding of GBM that could ultimately result in personalized therapies for groups of patients.^5,6^ Transcriptional profiling studies have revealed molecular subtypes of high-grade gliomas (grades 3 and 4) by Phillips *et al.*^7^ and of GBM by Verhaak *et al.*^8^ based on the preferential expression of genes characteristic of neural progenitor cells (proneural (PN)), neurons (neural (N)), proliferating cells and receptor tyrosine kinase activation (classical (CLAS)) or mesenchymal tissues (mesenchymal (MES)). A parallel comparison of these two studies revealed particularly strong agreement in the gene signatures associated with the PN and MES subtypes.^9^ Although still under evaluation, the different subtypes were reported to have prognostic value. GBMs of the MES subclass are predominantly primary tumors that originate *de novo* and were reported to exhibit a worse prognosis in comparison with the PN tumors.^7,10,11^ Better prognosis associated with the PN subtype may be because of the fact that a subset of PN tumors display mutations in the *isocitrate dehydrogenase 1* (*IDH1*) gene and display a glioma CpG island methylator phenotype (G-CIMP), both of which are favorable prognostic factors.^8,12^ In contrast, the MES tumors do not display G-CIMP, have a wild-type (WT) *IDH1* and possess alterations in *neurofibromatosis type-1* (*NF1*).^8,12^ A number of transcription factors, C/EBP-*β* (CCAAT-enhancer-binding protein-*β*) and STAT3 (signal transducer and activator of transcription 3) and more recently the transcriptional coactivator TAZ (transcriptional coactivator with PDZ-binding motif), have been identified as important regulators of the mesenchymal phenotype in GBM.^13,14^ However, in addition to these transcription factors it is conceivable that autocrine and paracrine interactions involving the microenvironment of GBM will also have a large impact on subtype status and tumor aggressiveness. Indeed, recently microglia cells were found to induce the mesenchymal status via a TNF*α*/NF-*κ*B (tumor necrosis factor-*α*/nuclear factor *κ*-light-chain-enhancer of activated B cells)-dependent manner that was associated with radioresistance.^15^

Transforming growth factor-*β* (TGF-*β*) plays a key role in tissue homeostasis and cancer, and in high-grade gliomas elevated TGF-*β* activity has been associated with poor clinical outcome.^16, 17, 18^ The secretion of TGF-*β* in GBM provides the tumor cells survival advantage by enhancing cell growth, migration, invasion, angiogenesis, immune suppression and stem cell properties.^17,18^ In preclinical GBM models, potent antitumor activity of TGF-*β* inhibition alone or in combination with radiochemotherapy has been demonstrated.^19,20^ These findings have spurred the development and testing of TGF-*β-*targeting agents in the patients with high-grade gliomas.^21, 22, 23^

TGF-*β* can activate a program called epithelial-to-mesenchymal transition (EMT) in epithelial cancers, such as breast, prostate and lung cancer, leading to enhanced migration and infiltration capacities of these cells, being a more common feature of mesenchymal cells.^24, 25, 26^ In an analogous way, it is conceivable that similar mechanisms will have a major impact on subtype status and tumor invasion in GBM. However, this notion has thus far remained unexplored.

In this study we examined the role of the TGF-*β* pathway as a determinant of mesenchymal differentiation in GBM. We identified TGF-*β* signaling as a strong inducer of mesenchymal transdifferentiation that was associated with enhanced tumor invasion in GBM. TGF-*β* may function locally in tumors to induce mesenchymal differentiation as a possible reaction to microenvironmental cues.

## Results

### Mesenchymal phenotype is associated with enhanced migratory capacity in GBM

**Figure 1:**
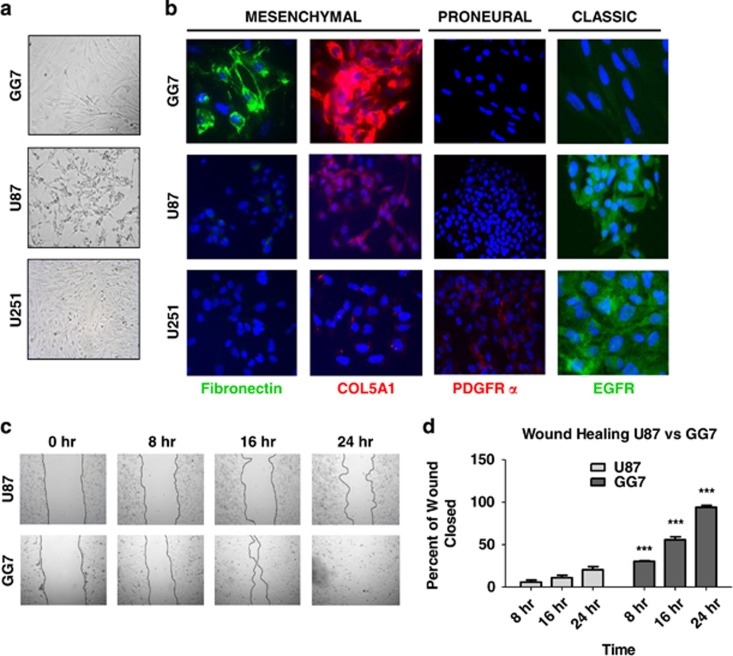
GBM cells with mesenchymal features have enhanced migratory capacity *in vitro*. (**a**) Phase contrast microscopic pictures ( × 10) of the newly generated GBM cell line GG7 along with the commercially available U87 and U251 cells. (**b**) Immunofluorescence analysis for mesenchymal (Fibronectin, COL5A1), proneural (PDGFR-*α*) and classic (EGFR) markers. (**c**) Wound healing assays comparing the migratory capacity of U87 *versus* GG7 cells at different time points. A representative experiment is shown. (**d**) Quantified data of wound healing assays where each data point represents the mean of at least three independent experiments±S.E.M. (***P<0.001 GG7 *versus* U87 for their respective time points)

First, the characteristics of a newly generated primary GBM monolayer cell line, named GG7, were compared with the well-established U87 and U251 GBM cell lines (Figure 1a). GG7 cells showed a spindle-shaped morphology when compared with the other GBM cell lines that had a more glial morphology. The expression levels of several neural stem cell/progenitor (Nestin and Vimentin), astrocytic (glial fibrillary acidic protein (GFAP)) and neuronal (*β*3 Tubulin) markers were examined. *β*3 Tubulin and Vimentin were present in all three cell lines, whereas expression of GFAP and Nestin was variable (Supplementary Figure 1). Based on previously published work,^8,13^ several markers reported as subtype specific were selected, that is, Fibronectin (MES), collagen 5A1 (COL5A1; MES), platelet-derived growth factor receptor-*α* (PDGFR*α*; PN) and epidermal growth factor receptor (EGFR; CLAS). Comparison of marker expression by immunofluorescence microscopy showed that GG7 has the highest levels of MES markers, whereas U87 and U251 have strong EGFR expression (Figure 1b). Next, we evaluated the migratory potential of U87 cells *versus* GG7 cells to determine the earlier reported notion that mesenchymal GBM cells have enhanced migratory capacity.^13^ In line with this, GG7 cells had a greater migratory capacity than U87 cells (Figures 1c and d). The possible contribution of proliferation toward enhanced migration seen in GG7 cells was ruled out as GG7 cells were found to divide even slower than U87 cells as determined by MTS (3-(4,5-dimethylthiazol-2-yl)-5-(3-carboxymethoxyphenyl)-2-(4-sulfophenyl)-2H-tetrazolium) assay (Supplementary Figure 2).

### TGF-*β* enhances the migratory capacity in GBM cells and promotes a mesenchymal shift *in vitro*

**Figure 2:**
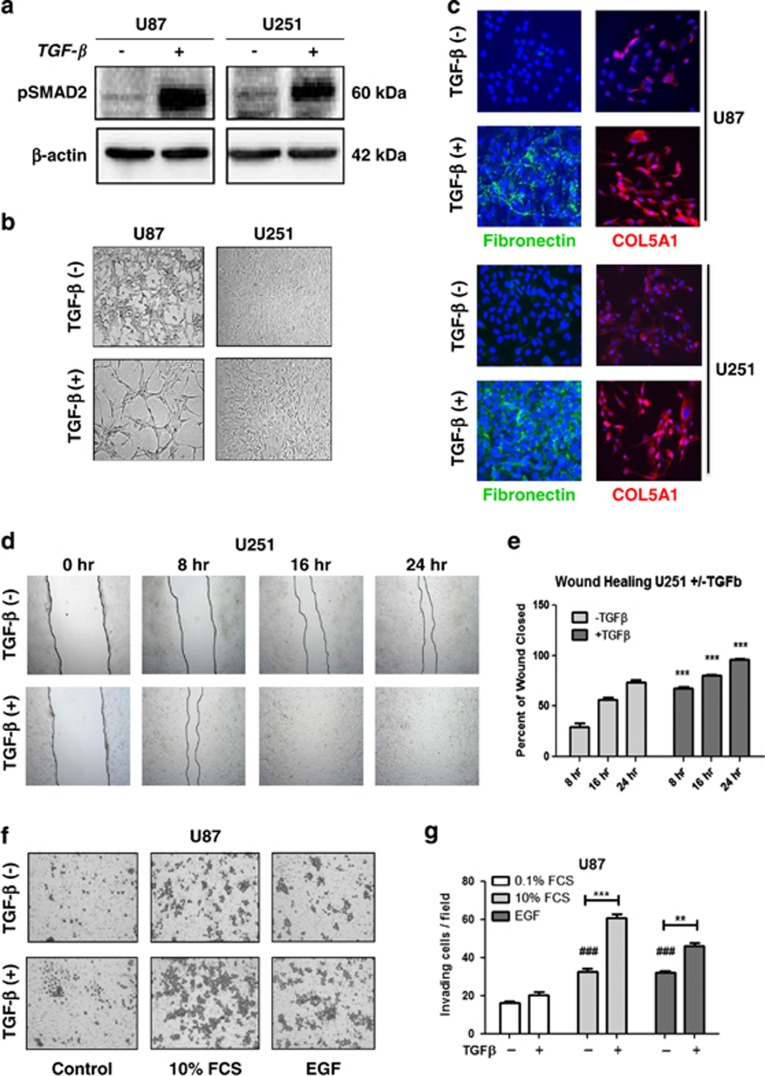
TGF-*β* induces mesenchymal transdifferentiation in GBM cells that is associated with enhanced migratory and invasive capacity. (**a**) Western blot showing the activation of TGF-*β* pathway as indicated by the phosphorylation of SMAD2. (**b**) Phase contrast microscopy at × 10 magnification showing TGF-*β*-induced changes in cellular morphology in U87 and U251 cells associated with spindle-shaped morphology and a more scattered growth pattern. (**c**) Immunofluorescence analysis depicting enhanced expression of the mesenchymal markers Fibronectin and COL5A1 following TGF-*β* exposure of U87 and U251 cells; images obtained at × 20 magnification. (**d**) A representative wound healing assay showing enhanced migratory capacity in U251 cells following exposure to TGF-*β* compared with the untreated group. Quantification of the wound closure capacity after 24 h of wound healing time (*n*=3) is shown in (**e**) (***P<0.001 U251 without TGF-*β versus* with TGF-*β* for their respective time points). (**f**) A representative Transwell collagen assay showing Coomassie blue-stained cells on the insert membranes, demonstrating enhanced invasive capacity following exposure to TGF-*β* when compared with untreated counterparts. Chemoattractants were 10% FCS or 0.1% FCS supplemented with EGF (50 ng/ml). Quantified data depicted in (**g**) and the bars represent the mean of in general three independent experiments measured in triplicate±S.E.M. (*P<0.05, **P<0.01 and ***P<0.001, U87 without *versus* with TGF-*β* for their respective groups; ^#^*P*<0.05, ^###^*P*<0.001, U87 10% FCS or EGF *versus* 0.1% FCS)

TGF-*β* has been reported as a potent inducer of EMT in epithelial cancers.^24,27,28^ In addition, TGF-*β* is also an important component of the GBM microenvironment.^17,18,20^ Taking these facts into consideration, the effects of TGF-*β* on U87 and U251 cells that have no or low mesenchymal marker expression were tested. Exposure to TGF-*β* (10 ng/ml) for 96 h activated phosphorylation of SMAD2 (Figure 2a) and led to a significant change in cellular morphology that was characterized by a more stretched and elongated appearance and an enhanced scattered growth pattern (Figure 2b). Concomitantly, TGF-*β* exposure enhanced the expression of mesenchymal markers Fibronectin and COL5A1, indicative of mesenchymal differentiation (Figure 2c).

Next, we examined the effect of TGF-*β*-induced mesenchymal transdifferentiation on the migration/invasion capacity of the GBM cells. U251 cells that were pretreated for 72 h with TGF-*β* showed enhanced migratory capacity when compared with the untreated control cells in wound healing assays (Figures 2d and e). To examine invasive properties, Transwell assays were used in which both TGF-*β*-treated and untreated cells were seeded on inserts coated with collagen, and were allowed to migrate toward two sets of chemoattractants, serum-free medium supplemented with EGF (100 ng/ml) or medium with 10% fetal calf serum (FCS). Enhanced invasive potential was observed in U87 cells following TGF-*β* treatment in comparison with the nontreated controls (Figures 2f and g). Together, these data indicate that TGF-*β* can induce mesenchymal transdifferentiation in GBM cells and promote their migratory and invasive potential *in vitro*.

### The TGF-*β* signaling inhibitor A8301 prevents induced mesenchymal differentiation and migration

**Figure 3:**
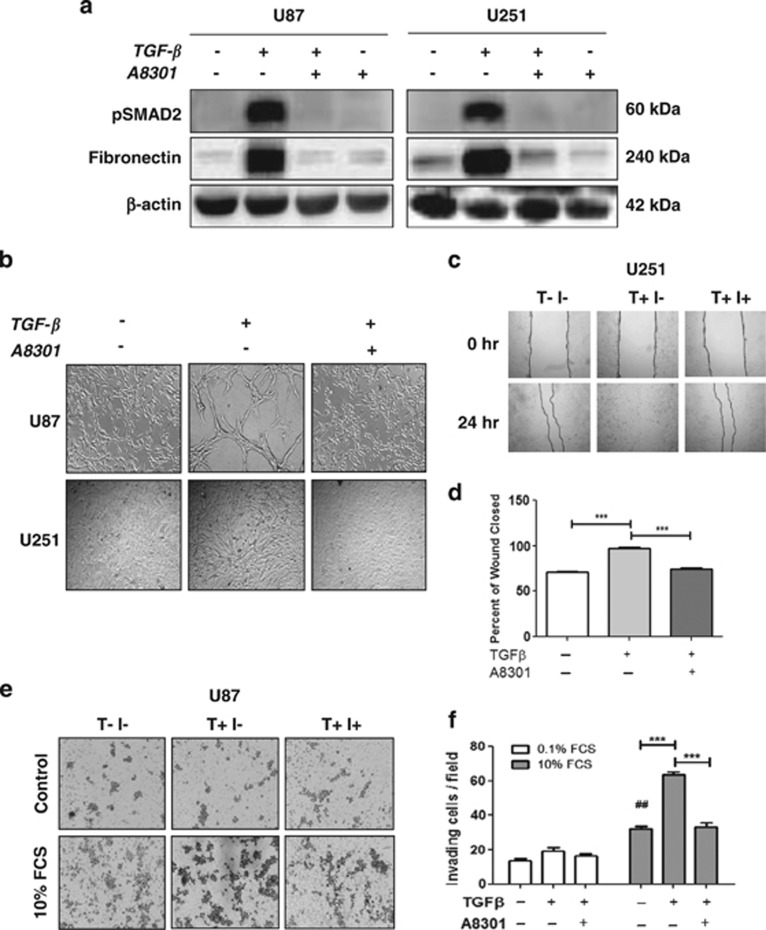
TGF-*β* signaling inhibitor A8301 prevents TGF-*β*-induced mesenchymal shift and enhanced invasive ability of GBM cells. (**a**) Western blots showing inhibition of TGF-*β*-induced SMAD2 phosphorylation and expression of the mesenchymal marker Fibronectin by A8301 (0.5 *μ*M). Representative blots are shown of *n*=3. (**b**) Phase contrast microscopic pictures at × 10 magnification of U87 and U251 cells treated with TGF-*β* in the presence or absence of A8301 in comparison with the untreated controls. The inhibitor prevented the phenotypic shift induced by TGF-*β*. (**c**) Wound healing assays showing reduced migration of TGF-*β*-treated U251 cells in the presence of A83-01. Quantification of the wound closure capacity (*n*=3, ***P<0.001) is shown in (**d**). (**e**) Transwell collagen assays showing reduced invasion of U87 cells toward 10% FCS following the addition of A83-01. Membranes were fixed and evaluated for cell numbers. A representative picture of the membranes showing invading U87 cells is shown in (**e**) and quantification of invasion assay is shown in (**f**) in which bars represent the mean of in general three independent experiments±S.E.M. (**P<0.01, ***P<0.001, U87 for 10% FCS group; ^##^*P*<0.01, for U87 10% FCS *versus* 0.1% FCS)

To further show the role of the TGF-*β* pathway for inducing a mesenchymal shift and migration/invasion in GBM, we employed A8301, a potent small-molecule blocker of the TGF-*β* type I receptors activin-like kinase 4 (ALK4, ALK5) and ALK7.^29^ The inhibitor was effective in blocking TGF-*β*-induced phosphorylation of SMAD2 and the upregulation of Fibronectin in U87 and U251 cells (Figure 3a). In addition, the phenotypic shift induced by TGF-*β* was completely prevented by A8301 (Figure 3b). Subsequently, we went on testing the efficacy of the inhibitor in blocking the migration and invasion capacity of the GBM cells. U251 cells treated with TGF-*β* in the presence of the inhibitor behaved in the same way as the nontreated controls, whereas wound closure was complete in U251 cells exposed to TGF-*β* alone (Figures 3c and d). Similar results were seen in Transwell assays, in which TGF-*β*-mediated invasion was inhibited by A8301 in U87 cells (Figures 3e and f).

### TGF-*β* enhances U87 xenograft tumor infiltration that is associated with increased mesenchymal properties and can be blocked by A8301

**Figure 4:**
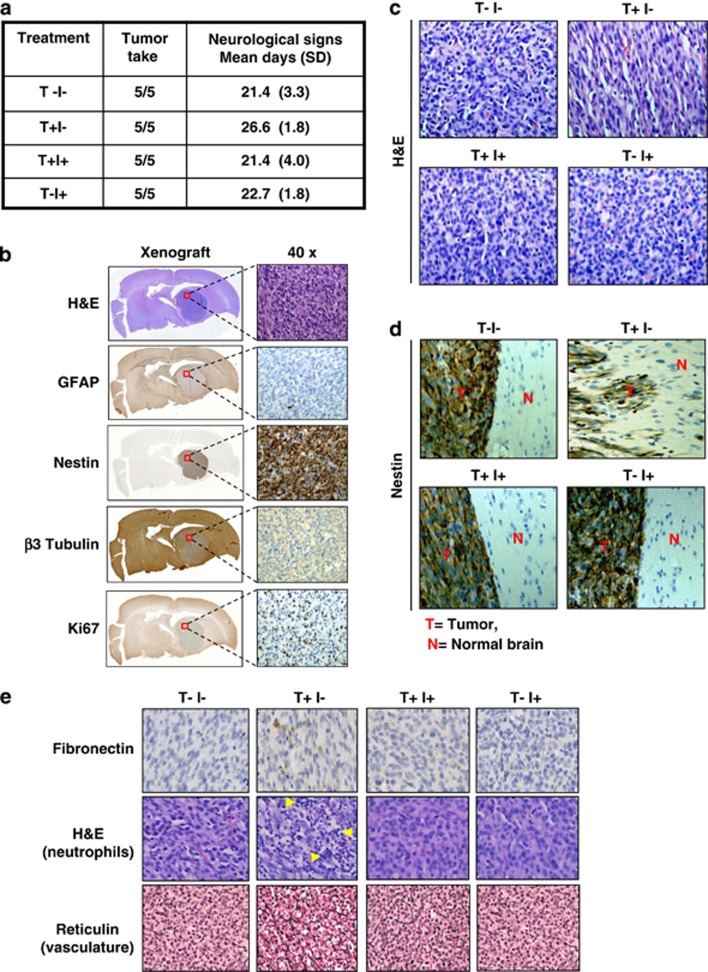
TGF-*β*-treated U87 cells show distinct tumor morphology and enhanced invasion after intracranial injection in NSG mice when compared with untreated cells, and can be prevented by cotreatment with A8301. (**a**) Overview of the experimental conditions used for intracranial transplantation. TGF-*β* (T) and A8301 (I), together with tumor take and the occurrence of neurological symptoms. (**b**) Immunohistochemical staining using the indicated markers of U87 xenografts. (**c**) Representative H&E staining of intracranial tumors derived from untreated U87 cells (T−I−), TGF-*β-*exposed cells (T+I−),TGF-*β*/A8301-exposed cells (T+I+) and cells exposed to A8301 alone (T−I+). TGF-*β*-treated tumor grafts showed cells with a more elongated/spindle-shaped cell morphology and appear more loosely packed when compared with nontreated, combined A8301 or A8301-alone-exposed cells. (**d**) Nestin-stained xenograft-normal brain parenchyma borders show enhanced invasion by groups or individual cells in TGF-*β*-stimulated U87-derived tumors (T+I−) when compared with the other conditions. (**e**) Expression of the mesenchymal marker Fibronectin was detected mainly in U87/TGF-*β* xenografts by immunohistochemistry. The T+I− tumors showed evidence of enhanced neutrophil infiltration (multinucleated cells, arrows) in H&E-stained specimens, and were also associated with increased vascularization and thicker basal membranes when compared with the other conditions visualized after Reticulin staining

In order to further study the relevance of TGF-*β* in promoting invasion in GBM, we employed an orthotopic U87 xenograft model in NSG mice. U87 cells were differently pretreated before injection into the striatum of the mouse brain: untreated (T−I−), treated with TGF-*β* in the absence of A8301 (T+I−), treated with TGF-*β* in the presence of A8301 (T+I+) and treated with A8301 alone (T−I+). Animals in all four experimental conditions developed tumor and were killed following presentation of neurological symptoms (Figure 4a). Immunohistochemical analyses of the xenografts revealed large ball-shaped tumor masses in the hematoxylin and eosin (H&E) stains. The obtained tumor was negative for GFAP and showed high Nestin expression and weak expression of *β*3 Tubulin, and a high proliferation rate as indicated by positive Ki67 staining (Figure 4b). In agreement with the staining pattern observed, the U87 cells used in establishing the intracranial tumor were also largely negative for GFAP; however, Nestin that was absent in the cell line was drastically enhanced in the tumor and the level of *β*3 Tubulin that was abundant in the cell line got reduced in the tumor, suggestive of influence of the microenvironment on expression of these markers (Supplementary Figure 1).

A deeper analysis of the H&E staining pattern revealed an elongated and loose tumor structure of TGF-*β*-treated U87-derived tumors (T+I−) in comparison with the other conditions (Figure 4c), thus resembling the above *in vitro* observations (Figure 2b). Moreover, examination of tumor boundaries in Nestin-stained samples revealed that cells of T+I− tumors infiltrated the adjoining brain, whereas tumors in the other three conditions had well-defined borders with apparently no infiltrating tumor cells (Figure 4d). The less compact growth pattern of TGF-*β*-treated cells may also explain the delayed presentation of neurological symptoms in animals of the T+I− group (Figure 4a), as this will likely reduce the rate at which intracranial pressure develops. Immunohistochemistry (IHC) also revealed that the T+I− group retained a considerable amount of cells expressing Fibronectin (Figure 4e). In 2 out of 5 animals in the T+I− group, further analysis of the tumor samples showed a strong infiltration of neutrophils into the tumor that was not observed in the other three groups (Figure 4e). Reticulin staining revealed a somewhat denser vasculature in the T+I− condition when compared with the other conditions (Figure 4e). Interestingly, neutrophils in the tumor microenvironment have been associated with angiogenesis in epithelial cancers.^30^ In order to test possible reversibility of the TGF-*β*-induced mesenchymal phenotype *in vitro*, U87 cells were cultured for 4 days with TGF-*β* and passaged 3 times for 12 days in the absence of TGF-*β* (supplementary Figures 3a and b). It was observed that following the withdrawal of TGF-*β*, the cells reverted back to their original morphology and lost the expression of Fibronectin. Notably, TGF-*β*-treated U87 cells mostly retained the acquired mesenchymal phenotype in mouse brains, implying a possible role of the microenvironment.

### ZEB1 mediates TGF-*β*-induced mesenchymal transition in GBM

**Figure 5:**
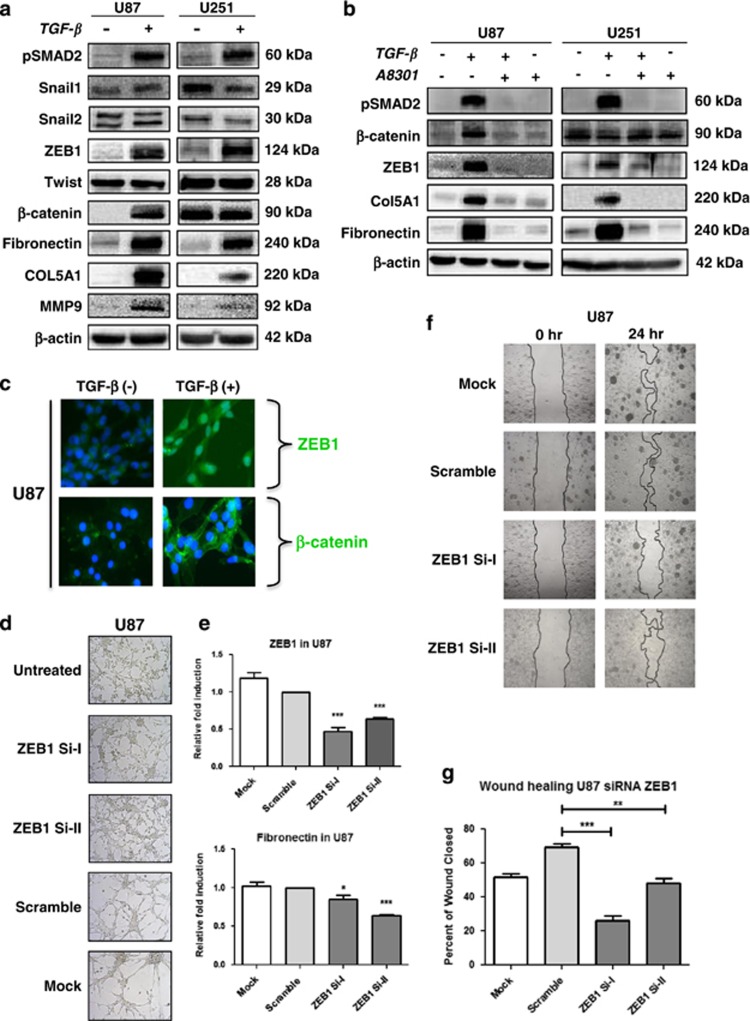
ZEB1 mediates TGF-*β*-induced mesenchymal transdifferentiation in GBM cells. (**a**) Western blots showing the effect of TGF-*β* administration on the expression of the indicated proteins, illustrating enhanced expression of the transcription factor ZEB1 in association with the upregulation of the mesenchymal markers Fibronectin, COL5A1 and MMP9 in U87 and U251 cells. The canonical Wnt signaling conferring transcription factor *β*-Catenin is also induced by TGF-*β* in U87 cells. Representative blots are shown of *n*=3. (**b**) A8301 prevents TGF-*β*-induced ZEB1 expression in GBM cells and inhibits *β*-Catenin accumulation in U87 cells. Consistently, induction of mesenchymal marker expression is prevented. Representative blots are shown of *n*=3. (**c**) Immunofluorescence microscopy ( × 40) showing accumulation and nuclear localization of ZEB1 in U87 cells. *β*-Catenin remains in the cytoplasm. (**d**) Phase contrast microscopy ( × 10) showing that TGF-*β*-induced acquisition of a mesenchymal morphology in U87 cells is prevented by siRNA-mediated silencing of ZEB1. Three different siRNAs directed against ZEB1 were used, of which the indicated two (siZEB1-I and siZEB1-II) were effective in blocking TGF-*β*-induced mesenchymal transdifferentiation when compared with siControl-transfected and mock-treated cells. (**e**) Expression levels were quantified with qRT-PCR and the bars represent the mean of in general three independent experiments measured in triplicate±S.E.M. (*P<0.05 and ***P<0.001, siZEB1-I and siZEB1-II *versus* scramble siRNA). (**f**) Wound healing assays showing reduced TGF-*β*-induced migratory activity upon silencing of ZEB1 using siZEB1-I or siZEB1-II. Quantified results of three independent results are shown in (**g**) (**P<0.01 and ***P<0.001, siZEB1-I and siZEB1-II *versus* scramble siRNA)

To obtain insight into the mechanisms underlying TGF-*β*-induced mesenchymal shift in GBM, the expression of various transcription factors associated with EMT, such as Snail1, Snail2/Slug, ZEB1 (zinc-finger E-box-binding homeobox 1), Twist and *β*-Catenin, was examined in U87 and U251 cells. Of these transcription factors, only TGF-*β*-dependent upregulation of ZEB1 and *β*-Catenin was observed, concurrent with the occurrence of Fibronectin, COL5A1 and metalloproteinase-9 (MMP9) (Figure 5a). Of note, we detected the 124 kDa form of ZEB1 and not the larger ~200 kDA form, both of which are known to be specific for ZEB1.^31^ The inhibitor A8301 prevented the upregulation of ZEB1 in U87 and U251 cells, of *β*-Catenin in U87 cells as well as of Fibronectin and COL5A1 in both the cell lines, thus potentially linking ZEB1 and *β*-catenin to TGF-*β*-induced mesenchymal transition (Figure 5b).

Both ZEB1 and *β*-Catenin have been reported as mediators of mesenchymal transition in other tumor types.^24,32^ To examine their role in mesenchymal transition in GBM, the subcellular localization of both transcription factors was examined using immunofluorescence staining. U87 cells exposed to TGF-*β* showed nuclear ZEB1, whereas enhanced *β*-Catenin expression was largely seen in the cytoplasm with no detectable nuclear translocation (Figure 5c). Furthermore, knockdown and chemical inhibition of *β*-Catenin revealed no effect on TGF-*β*-induced mesenchymal transdifferentiation in U87 cells, indicating no essential role for this protein (data not shown). On the other hand, time course experiments showed that ZEB1 accumulation coincided with enhanced expression of COL5A1 and Fibronectin in U87 cells, further suggesting a role of ZEB1 in mesenchymal transdifferentiation (Supplementary Figure 4). Indeed, small interfering RNA (siRNA)-dependent silencing of ZEB1 using two different selective siRNAs in U87 cells resulted in inhibition of the TGF-*β*-induced morphological shift, as shown for U87 in Figure 5d. As controls, the ZEB1 siRNAs effectively reduced ZEB1 transcript levels when compared with nonspecific siRNAs, and subsequently also reduced induction of Fibronectin transcripts, as determined by quantitative real-time PCR (qRT-PCR; Figure 5e). As an additional control, the siRNA-dependent downregulation of ZEB1 and the consequent inhibition of Fibronectin induction upon TGF-*β* treatment was also evident at the protein level (Supplementary Figure 5). Wound healing assays showed that ZEB1 silencing reduced TGF-*β*-induced migration in U87 cells (Figures 5f and g). Finally, the exposure of mesenchymal GG7 cells to TGF-*β* could induce a further increase in ZEB1 and COL5A1 expression and migratory potential (Supplementary Figures 6a–c). The possible involvement of differences in proliferation rates in these experiments were ruled out by finding even a reduction in proliferation after TGF-*β* treatment in these cells (Supplementary Figure 2). Together, these data indicate a crucial role of the TGF-*β–*ZEB1 axis in mediating mesenchymal transdifferentiation and enhancement of the invasive capacity of GBM cells.

### PN GBM neurospheres can acquire mesenchymal properties upon intracranial implantation

**Figure 6:**
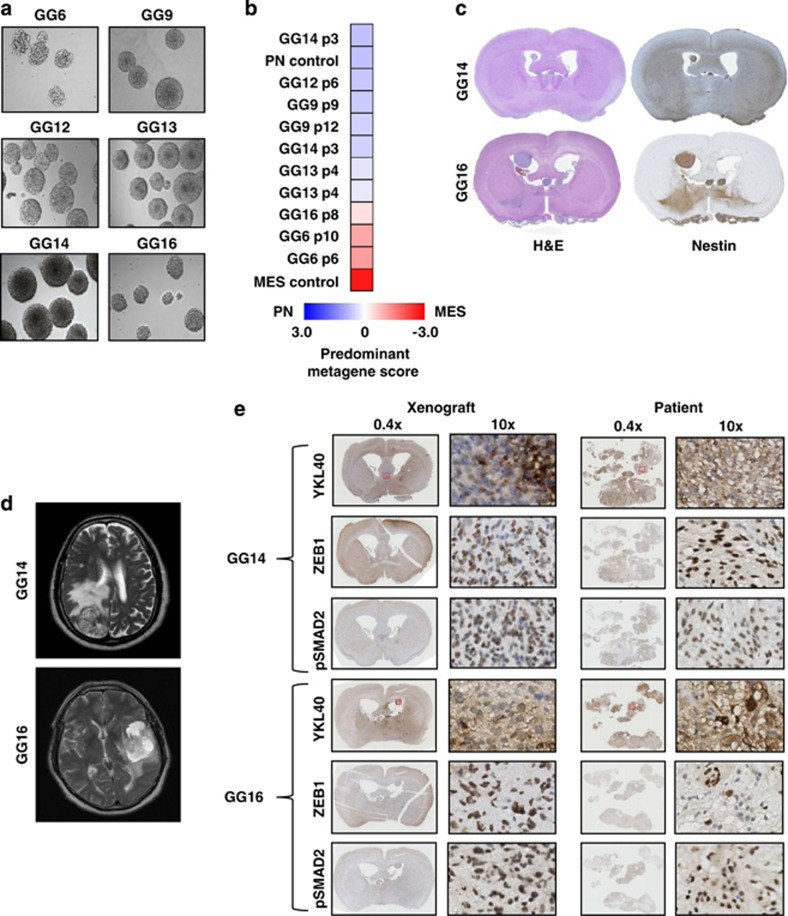
Characterization of patient-derived GBM neurospheres indicates that PN cells can gain mesenchymal features upon implantation in mice. (**a**) Morphology of six patient-derived neurospheres using phase contrast microscopy, images obtained at × 10 magnification. (**b**) Heatmap of the predominant signature of the indicated panel of GBM neurospheres. A qRT-PCR-based PN/MES metagene analysis was performed on each sample allowing calculation and comparison of *z*-scores. Blue shades indicate PN, and red a MES signature. Included were samples derived from previously subtyped GBM patients representing PN and MES signatures. For some neurosphere cultures different passage numbers (p) were included. (**c**) H&E- and Nestin-stained sections of tumor grafts derived from the indicated neurospheres showing tumor growth and dissemination in the mice brains. (**d**) T2 MRI images of patients from which GG14 and GG16 cell lines were derived showing infiltrative GBM with massive edema and necrosis. (**e**) Immunohistochemical staining comparing the expression of YKL40, ZEB1 and pSMAD2 in GG14 and GG16 xenografts in parallel with their corresponding patient material

We continued by investigating whether the GBM subtype of isolated primary GBM neurospheres may also show variability in differentiation status and tumor properties. Therefore, six newly generated primary neurospheres named GG6, GG9, GG12, GG13, GG14 and GG16 (Figure 6a) were first characterized for the expression of MES and PN markers using a previously described qRT-PCR-based metagene analysis with sets of four PN- and four MES-specific genes.^15^ The analysis identified GG6 and GG16 to be mostly mesenchymal, and GG9, GG12, GG13 and GG14 to have enhanced proneural gene expression (Figure 6b). Overall, the subtype was maintained at different passage numbers, although we generally used passage numbers below 10 in our experiments.

**Figure 7:**
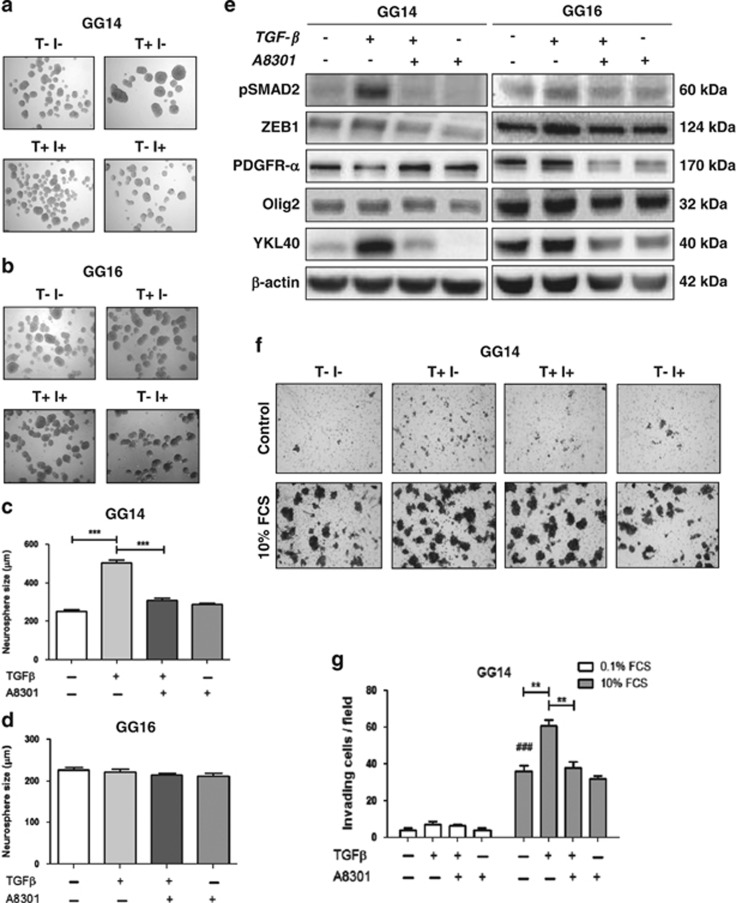
Neurospheres demonstrate variability in acquiring mesenchymal properties following TGF-*β* treatment. (**a** and **b**) GG14 (PN) neurospheres exposed to TGF-*β* show enhanced proliferation as indicated by increased neurospheres sizes that could be prevented by the administration of A8301. This effect was not observed in GG16 (MES) neurospheres (**c** and **d**). Data represent the means±S.E.M. of 3 independent experiments where 30 neurospheres were included in each experiment (***P<0.001). (**e**) Western blots showing the effect of TGF-*β* treatment on GG14 and GG16 in terms of the levels of the proneural markers (PDGFR-*α*, OLIG2) and mesenchymal marker (YKL40) along with ZEB1 and pSMAD2. (**f**) Representative Transwell membranes showing enhanced infiltrative capacity of TGF-*β*-treated GG14 neurospheres; quantification (*n*=3) is shown in (**g**) (**P<0.01 and ***P<0.001, ^###^*P*<0.001, for GG14 control cells 0.1% FCS *versus* 10% FCS)

Based on the metagene analysis, we selected GG14 and GG16 cells as being most divergent for comparing tumor growth and invasive behavior of MES and PN GBM cells. Upon intracranial implantation in NSG mice, both formed equally effective invasive tumors, with GG14 producing tumors that resemble gliomatosis cerebri (Figure 6c). Inspection of magnetic resonance imaging (MRI) obtained from the corresponding patients also showed similar tumor growth patterns with massive edema and necrosis (Figure 6d). Furthermore, IHC analyses of the GG14 and GG16 xenografts and corresponding patient material revealed that both tumors similarly express YKL40, the established mesenchymal marker^7^ (Figure 6e), contrasting the much lower expression level of this marker in GG14 cells in culture (Supplementary Figure 7a and Figure 7e). In addition, both xenografts and patients tumor tissues displayed pSMAD2 and ZEB1 staining (Figure 6e). We also observed comparable expression patterns of Nestin, PDGFR-*α*, oligodendrocyte transcription factor (OLIG2), and EGFR between xenografts and the corresponding patient material (Supplementary Figure 7b). Thus, the subtype of the cultured neurospheres does not necessarily predict tumor growth characteristics in mice and patients, and importantly PN cells can acquire mesenchymal properties.

### GG14 and GG16 cells respond differently to TGF-*β*

We then proceeded by examining the invasive potential of GG14 PN and GG16 MES neurospheres in Transwell assays. GG16 cells had a somewhat stronger invasive capacity than GG14 cells (Supplementary Figures 8a and b). Next, GG14 and GG16 neurospheres were treated with TGF-*β* in the presence or absence of A8301. We noted that TGF-*β* treatment for 4 days significantly increased the size of the GG14 neurospheres and this effect was prevented by A8301. However, TGF-*β* did not show an effect on GG16 neurosphere sizes indicative of the involvement of yet unknown cell-specific determinants (Figures 7a–d). The effect of TGF-*β* on GG14 neurosphere size is in line with a previous report showing TGF-*β-*dependent enhancement of GBM neurosphere growth.^33^ Moreover, TGF-*β* treatment induced phosphorylation of SMAD2 and enhanced ZEB1 expression in GG14 that was associated with a reduction of PDGFR-*α*, whereas OLIG2 expression remained the same, and a gain of YKL40 expression. GG16, on the other hand, did not respond to TGF-*β* treatment with respect to these markers (Figure 7e). Furthermore, TGF-*β* exposure enhanced GG14 cell invasion as determined in Transwell assays (Figures 7f and g).

### Overlapping pSMAD2, ZEB1 and YKL40 expression in patient material

**Figure 8:**
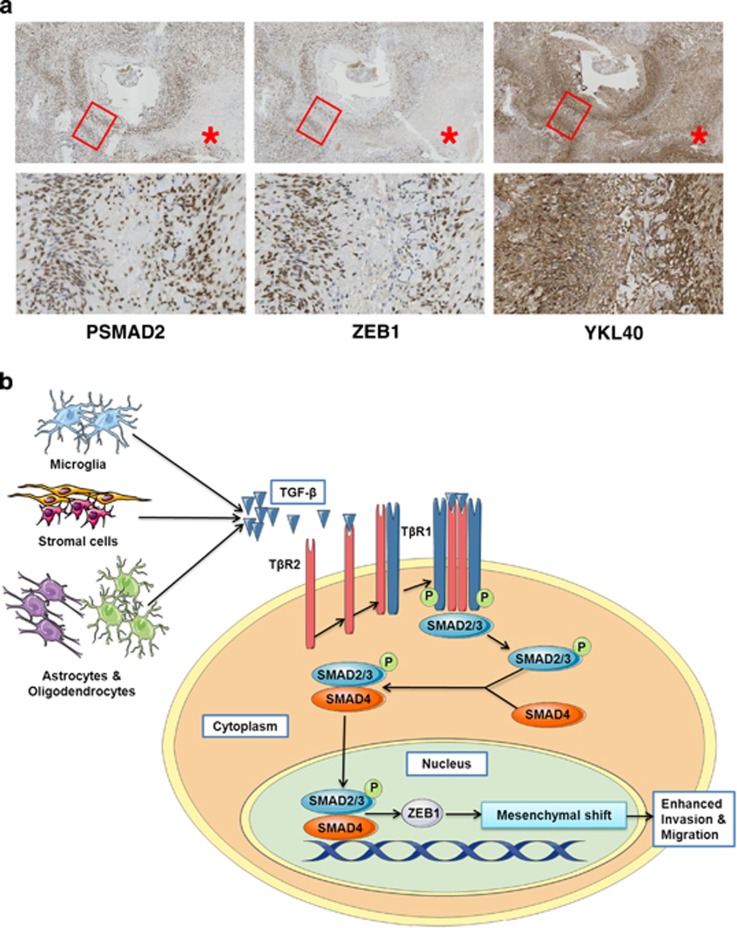
Local/regional mesenchymal transition detected in GBM patient material. (**a**) Immunohistochemical staining for pSMAD2, ZEB1 and YKL40 in consecutive sections detects overlapping expression patterns of pSMAD2, ZEB1 and YKL40 in perivascular areas in GBM patient tissue. (**b**) Model illustrating TGF-*β*-induced mesenchymal transition in GBM that is mediated by pSMAD2 and ZEB1. TGF-*β* may be produced by tumor cells, microglia or other stromal cells leading to a local induction of mesenchymal properties in GBM. This contributes to heterogeneity in GBM subtype

Finally, in order to obtain further evidence for the occurrence of TGF-*β*-dependent mesenchymal transition in GBM, we performed IHC for pSMAD2, ZEB1 and YKL40 detection in serial slices from GBM patient material. Frequently, we observed an overlapping pattern of zonal expression of these markers in perivascular areas in the patient material (Figure 8a).

Together, this gives a clear indication on the role of either autocrine or paracrine produced TGF-*β* in inducing mesenchymal transition as schematically depicted in Figure 8b. This will contribute to heterogeneity in GBM.

## Discussion

GBMs of the MES subclass have been linked with high aggressiveness and resistance to treatment, whereas patients with a PN signature were reported to perform better in the clinic with respect to survival and treatment responses.^7,8,11^ Interestingly, in some patients with recurrent disease, a shift from a PN tumor into a MES subtype was observed and is assumed to be induced by therapy.^7^ However, the boundaries between different GBM subtypes appear less sharp, and recently the presence of a number of different GBM subtypes within the same tumor was demonstrated by comparing transcriptional profiles of different spatially distinct GBM fragments in one patient.^34^ As proposed, this may reflect coexisting cell lineages within the same tumor. However, it is also likely that tumor cell–microenvironment interactions will have an impact on subtype status and thus tumor aggressiveness in GBM, similar to what has been found for epithelial tumors.^35^ In this report, we provide evidence for this hypothesis by demonstrating that TGF-*β*, well known for its ability to induce EMT in epithelial cancers, can induce a ZEB1-dependent mesenchymal transdifferentiation in GBM. TGF-*β* is known to be abundantly present in the tumor microenvironment of GBM and has been linked to multiple processes associated with GBM, such as angiogenesis, invasion/migration, immunosuppression and stemness.^17,18^ We show that this mesenchymal shift in GBM is associated with enhanced migration and invasion capacity of tumor cells in cell culture and intracranial mouse models. Treatment with the TGF-*β* signaling inhibitor A8301 as well as ZEB1 knockdown prevented the acquisition of mesenchymal marker expression and morphological changes, thus linking mesenchymal differentiation in GBM with enhanced tumor cell invasion through the TGF-*β–*ZEB1 axis. Interestingly, and in line with our observations of the importance of ZEB1, is a recent report showing that the ZEB1-mir-200 feedback loop is involved in invasion, chemoresistance and tumorigenesis in glioblastoma, regulating the expression of, among others, methylguanine methyltransferase (MGMT) and CD133.^36^ In addition, we also observed differences in tumor vasculature and enhanced infiltration of neutrophils in TGF-*β*-treated implanted U87 cells, although not consistently. The TGF-*β*-induced mesenchymal shift was mainly detected in GBM cells with low or absent mesenchymal marker expression; however, GG7 GBM cells with already elevated mesenchymal marker expression also showed enhanced mesenchymal properties and elevated ZEB1 expression and migration following TGF-*β* treatment.

When comparing the tumorigenic potential of GG14 PN and GG16 MES neurospheres upon intracranial injection in mice, we did not observe clear differences. Both formed invasive tumors with extensive disseminated growth of GG14. In line with this, MRI scans of the corresponding GG14 and GG16 patients showed similar growth properties of the tumors. Notably, IHC analyses of the xenografts and the corresponding patient material also showed heterogeneous expression of proneural and mesenchymal markers, along with pSMAD2 and ZEB1 staining. Activation of this pathway and the acquisition of mesenchymal marker expression could involve cues from the microenvironment, including murine TGF-*β*, as TGF-*β* is highly homologues in higher vertebrates and cross-species activity was demonstrated previously.^37,38^ On the other hand, *in vitro* assays identified differences between GG14 PN and GG16 MES cells. GG16 was somewhat more invasive in Transwell assays but did not show an apparent increase in invasiveness after TGF-*β* treatment. In contrast, the invasive capacities as well as growth properties of GG14 cells were enhanced by TGF-*β* exposure. In agreement to this finding, TGF-*β* could induce pSMAD2 and ZEB1 expression in GG14, whereas in GG16 cells, considerable expression of these proteins could already be detected. Apparently, GBM cells have an inducible or already activated TGF-*β*–ZEB1 pathway in a cell-specific way. A possible autocrine activation of this pathway appears not necessarily to be involved as A8301 addition to both GG7 and GG16 cells did not affect pSMAD2 or ZEB1 levels. The molecular mechanism involved in inherent activation of the pSMAD2 and ZEB1 remains to be elucidated. Regardless of this, our results show that TGF-*β*-induced signaling can lead to a gain in mesenchymal marker expression and invasive behavior in GBM.

TGF*β*-dependent activation of ZEB1 has been reported in other tumor types. For example, recently non-CSCs of human basal breast cancer were shown to be able to switch to a CSC state as a result of ZEB1 activation.^39^ Plasticity of these cells involved a bivalent chromatin configuration of the ZEB1 promoter, allowing an effective transcriptional response to microenvironmental signals as was shown toward TGF*β* in this model. In GBM it has been reported that TGF*β* enhances self-renewal potential in glioma-initiating cells through the secretion of leukemia inhibitory factor (LIF).^33^ Whether the TGF*β*-induced mesenchymal status in GBM, as we report here, leads to enhanced stemness remains to be investigated.

Recently, microglia-derived TNF*α* was reported to induce a mesenchymal state in a subset of PN GBM neurospheres through activation of NF-*κ*B.^15^ A correlation was found between MES signature, CD44 expression and NF-*κ*B activation and a poor response to radiotherapy and shorter survival. Our finding that TGF*β* can induce mesenchymal transition provides another secretory factor that is able to trigger mesenchymal differentiation in GBM, thus contributing to tumor heterogeneity and enhanced tumor aggressiveness. Therapeutic strategies aimed at preventing mesenchymal transition, either at the level of the initiating signal or downstream that is, ZEB1, offer attractive strategies for the treatment of a subset of GBM patients.

## Materials and Methods

### Cell culture and treatments

The human GBM cell line U87 was obtained from the American Type Culture Collection (ATCC, Manassas, VA, USA) and U251 was obtained from the CLS Cell Lines Service GmbH (Eppelheim, Germany). Monolayer GG7 cells and the neurospheres GG6, GG9, GG12, GG13, GG14 and GG16 were newly generated from human GBM surgical samples. These primary materials were pathologically confirmed as GBM, GG16 being giant-cell GBM. Primary material was obtained after approval and following the ethical guidelines of the Institutional Review Board of the UMCG. Freshly resected tumor material was washed 5 times in cold PBS followed by mechanical dissociation and incubation in trypsin (Gibco Life Technologies, Bleiswijk, The Netherlands) at 37°C for 15 min. After incubation the tissue was repeatedly pipetted and the cell suspension was filtered through a 70 *μ*m cell strainer (BD Falcon, Franklin Lakes, NJ, USA) to obtain single cells. GG7 cells were pelleted by centrifugation at 1200 r.p.m. for 10 min and resuspended in Dulbecco's modified Eagle's medium high glucose (DMEM-Hg) (Gibco Life Technologies) medium supplemented with 10% FCS and 2% penicillin(pen)/streptomycin(strep) (Gibco Life Technologies) and maintained for two passages after which the concentration of pen/strep was reduced to 1%. GG7 and U251 were grown in regular cell culture flasks, whereas U87 required precoating of the flasks with 1% gelatine from porcine skin (Sigma-Aldrich Chemie BV, Zwijndrecht, The Netherlands). Monolayers were maintained in DMEM-Hg supplemented with 10% FCS and 1% pen/strep. The neurospheres were generated following propagation in Neurobasal A-Medium (Gibco Life Technologies) supplemented with 2% B27 supplement (Gibco Life Technologies), 20 ng/ml EGF (R&D Systems, Abingdon, UK), 20 ng/ml basic fibroblast growth factor (bFGF; Merck Millipore, Billerica, MA, USA), 1% pen/strep and 1% L-glutamine (Gibco Life Technologies). The neurospheres were characterized for the expression of neuronal stem cell markers, including SOX2 (sex determining region Y-box 2), OCT-4 (octamer-binding transcription factor 4), OLIG2, Nestin and Musashi, and differentiation markers GFAP and *β*3Tubulin by RT-PCR, western blotting or immunofluorescent microscopy (not shown).

Cell lines were maintained at 37°C in a humidified atmosphere with 5% CO_2_. When indicated, cells were treated with TGF-*β* (10 ng/ml; PeproTech, London, UK) and/or the small-molecule inhibitor of the TGF-*β* receptor, A 8301 (Axon Medchem, Groningen, The Netherlands). The inhibitor was added at a concentration of 0.5 *μ*m 2 h before the addition of TGF-*β*.

### Migration and invasion assays

The migratory capacity of cells was determined by wound healing assays. Briefly, 2 × 10^5^ cells where seeded on poly-L-Lysine (Sigma-Aldrich)-coated six-well plates in culture medium; upon confluency a scratch was made using a P10 pipette tip. The rate of wound closure was monitored at different time points under a microscope and quantified using ImageJ software (NIH, Bethesda, MD, USA). The invasion potential was determined on collagen-coated Transwell inserts with 8 *μ*m pore size (Becton Dickinson BV, Breda, The Netherlands). For this, cells were trypsinized and 150 *μ*l of a cell suspension containing 2.5 × 10^4^ cells (U87) or 5 × 10^4^ cells (GG14 and GG16) were added to Transwells in triplicates per condition. Then, 10% FCS or 0.1% FCS with 100 ng/ml EGF was added to the lower wells as chemoattractants. Cells that migrated/invaded and appeared on the bottom surface of the Transwell insert membrane were fixed with 75% methanol/25% acetic acid for 20 min and stained with 0.25% Coomassie blue in 45% methanol/10% acetic acid followed by washing with demi water. The membranes were subsequently cut out and mounted on microscopic slides for quantification. Representative pictures of the membranes with cells were acquired at × 10 magnification and the total number of cells on 10 individual fields per membrane were counted; average numbers and S.D. of invading cells for every condition were calculated.

### Immunofluorescence microscopy

Cells cultured on poly-L-lysine (Sigma-Aldrich)-coated coverslips were fixed for 10 min using 4% formaldehyde or 100% methanol. After 3 times washing with cold PBS, cells were permeabilized with 0.1% Triton (Sigma-Aldrich) in PBS, washed again with PBS followed by a blocking step for 1 h with PBS+0.1% Tween-20 (Sigma-Aldrich), 2% bovine serum albumin (BSA; PAA Laboratories GmbH, Colbe, Germany) and 1 : 50 dilution of normal goat serum (Dako Denmark A/S, Glostrup, Denmark). Subsequently, cells were incubated with the indicated primary antibodies at room temperature for 1.5 h. Primary antibodies used were as follows: purified mouse anti-Fibronectin (1 : 50; 610077; BD Transduction Laboratories, San Jose, CA, USA), anti-COL5A1 (1 : 200; sc-20648; Santa Cruz Biotechnology, Santa Cruz, CA, USA), anti-PDGFR-*α* (1 : 500; ab61219; Abcam, Cambridge, UK), anti-EGFR (Merck Millipore), anti-ZEB1 (1 : 50; sc-10572; Santa Cruz Biotechnology Inc.), Anti-Active- *β*-Catenin (1 : 100; 05-665; Merck Millipore). After 3 times washing with PBS, slides were incubated for 1 h with the appropriate secondary antibodies: goat anti-Mouse Alexa488 (1 : 200, Life Technologies), Donkey anti-Human Alexa488 (1 : 200), Donkey anti-goat Alexa488 (1 : 200) or Goat anti-Rabbit IgG Antibody, Cy3 conjugate (1 : 400; AP132 C; Merck Millipore). Hoechst (Sigma H6024) staining was performed for 5 min followed by mounting the coverslips with Kaisers glycerin (Merck Millipore). Cells were examined by fluorescent microscopy (Leica DM6000, Leica Microsystems GmbH, Mannheim, Germany) and images were captured using Leica DFC360 FX camera.

### Immunohistochemistry

Formalin-fixed, paraffin-embedded 5 *μ*m thick tissue sections were mounted on microscope slides and dried overnight at 55°C. Tissue sections were deparaffinized in xylol and rehydrated in graded series of ethanol and stained with H&E. Sections were subjected to microwave pretreatment either in pH 6.0 citrate buffer when stained for OLIG2 (rabbit polyclonal Ab; IBL-International, Toronto, ON, Canada), PDGFR-*α* (rabbit polyclonal Ab; Santa Cruz Biotechnology Inc.), EGFR (mouse monoclonal Ab; Monosan, Uden, The Netherlands), Nestin (mouse monoclonal Ab; Santa Cruz Biotechnology Inc.), phospho-SMAD2 (rabbit polyclonal Ab; Cell Signaling, Danvers, MA, USA), *β*-III-Tubulin (mouse monoclonal Ab; Merck Millipore), CD44 (monoclonal Ab; BioLegend Inc., San Diego CA, USA), GP39 (polyclonal Ab; Santa Cruz Biotechnology Inc.), ZEB1 (polyclonal Ab; Sigma-Aldrich Chemie BV) or in Tris-EDTA pH 9.0 buffer for staining Ki-67 (mouse monoclonal Ab; Dako). No antigen retrieval was required for GFAP staining (rabbit polyclonal Ab; Dako). Before staining, sections were treated with 0.3% H_2_O_2_ for 30 min and blocked for 1 h with 2% BSA to reduce nonspecific primary antibody binding. As negative controls, primary antibodies were omitted. After incubation with primary antibody at 4°C overnight, suitable secondary antibodies conjugated to peroxidase (Dako) and appropriate tertiary antibodies conjugated to peroxidase (Dako) were used. Staining was visualized by 3,3′-diaminobenzidine and sections were counterstained with hematoxylin and mounted. Images of relevant sections were acquired using a Leica DFC 420C digital camera (Leica Microsystems), connected to a Leica DM 3000 microscope, using Leica Application Suite software. Images were also acquired with TissueFaxs/Zeiss AxioObserver Z1 Microscope System (TissueGnostics GmbH, Vienna, Austria).

### Western blotting

In brief, cells were harvested, washed with cold PBS and lysed with M-per mammalian protein extraction agent (Thermo Fisher Scientific, Waltham, MA, USA) supplemented with 1% protease inhibitor (Thermo Fisher Scientific) and 1% phosphatase inhibitor (Thermo Fisher Scientific) for 1.5 h on ice. Next, the suspension was centrifuged for 10 min at 14000 r.p.m. at 4°C and the supernatant was taken for determining protein concentrations using a Bradford assay (Bio-Rad, Hercules, CA, USA). Then, 25–50 *μ*g of proteins per sample per lane were loaded for sodium dodecyl sulfate-polyacrylamide gel electrophoresis (SDS-PAGE). Proteins were then transferred to PVDF membrane (Millipore IPVH00010 0.45 *μ*m). For staining, the membrane was blocked for 1 h at room temperature (RT) with 5% milk in TBST (20 mmol/1 Tris-HCL (pH 8.0), 137 mmol/l NaCl and 0.1% Tween-20) or with 5% BSA in TBST for phospho-proteins. Primary antibodies were incubated overnight at 4°C. Primary antibodies used were: Polyclonal Rabbit anti-GFAP (1 : 1000; N1506; Dako), anti-*β*ІІІ Tubulin antibody (1 : 2000; ab76287; Abcam), Nestin (1 : 500; sc-23927; Santa Cruz Biotechnology, Inc.), Vimentin (1 : 500; sc-373717; Santa Cruz Biotechnology Inc.), Phospho-SMAD2 (1 : 1000; #3108; Cell Signalling), SNAI1/Snail1 (1 : 500; sc-10433; Santa Cruz Biotechnology Inc.), SLUG/SNAIL2 (1 : 500; sc-166476; Santa Cruz Biotechnology Inc.), ZEB1 (1 : 500; sc-81428; Santa Cruz Biotechnology Inc.), ZEB1 (1 : 500; sc-10572; Santa Cruz Biotechnology Inc.), Anti-Twist antibody (1 : 1000; ab50581; Abcam), Anti-Active-*β*-Catenin (1 : 1000; 05-665; Merck Millipore), Anti-MMP9 antibody (1 : 5000; ab76003; Abcam), purified mouse anti-Fibronectin (1 : 2500; 610077; BD Transduction Laboratories) and COL5A1 (1 : 2000; sc-20648; Santa Cruz Biotechnology, Inc.). After incubation, membranes were washed with TBST, and reprobed with appropriate horseradish peroxidase (HRP)-conjugated secondary antibodies, anti-mouse immunoglobulin G (IgG), anti-rabbit IgG or anti-goat IgG) (Dako) for 1 h at RT. Proteins were visualized using Amersham Biosciences enhanced chemiluminescence (ECL) detection system (GE Healthcare, Little Chalfont, UK).

### Intracranial injection mouse model

U87 cells untreated or pretreated with TGF-*β* (T) for 96 h in the presence or absence of the TGF-*β* inhibitor (I) A8301 (Axon Medchem) added 2 h before TGF-*β* addition were prepared for intracranial injection in NOD SCID gamma mice (NOD.Cg-*Prkdc*^*scid*^ *Il2rg*^*tm1Wjl*^/SzJ)/NSG mice) obtained as inbred strains from the Central Animal Facility (Groningen, The Netherlands). A total of 5 animals per condition were used, and 3 × 10^5^ GG5 cells (1 × 10^5^/*μ*l PBS) were injected in the striatum of the animals using a stereotactic frame. Then, 5 × 10^5^ cells of GG14 and GG16 were injected (3 animals/cell line) to determine tumorigenicity and invasive growth. Mice were monitored and killed when they presented with neurological signs or after being 6 months in the experiment, following which the brains were harvested and fixed in 4% paraformaldehyde for 48 h and embedded in paraffin and prepared for IHC. These experiments were approved by the committee for Animal care and conducted in compliance with the Animal Welfare Act Regulations.

### Short interfering RNA treatment

Validated Stealth RNAi (OriGene SR304746, Rockville, MD, USA) specific to ZEB1 and *β*-Catenin (Cell Signaling # 6225) was transfected into U87 cells by using Lipofectamine RNAiMAX (Thermo Fisher Scientific) according to the manufacturer's protocol. Trilencer-27 Universal scrambled negative control siRNA (OriGene SR30004) was used as negative control. The downregulation of ZEB1 and *β*-Catenin was examined using RT-PCR.

### Quantitative real-time PCR

Total RNA from siRNA-transfected and mock-treated GBM cell lines was isolated using RNeasy mini kit (Qiagen, Hilden, Germany) according to the instruction of the manufacturer. RNA was analyzed quantitatively using Nanodrop (Nanodrop Technologies, Rockland, DE, USA). Total RNA (1 *μ*g) was reverse transcribed into cDNA by a RNase H+ reverse transcriptase using iScript cDNA synthesis kit (Bio-Rad) according to the manufacturer's instructions. The cDNAs were stored at −20°C. RT-PCR was performed in a ABI PRISM 7900HT Sequence Detector (Applied Biosystems, Foster City, CA, USA) with the iTaq SYBR Green Supermix with Rox dye (Bio-Rad) and amplification was performed with the following cycling conditions: 5 min at 95°C, and 40 two-step cycles of 15 s at 95°C and 25 s at 60°C. The reactions were analyzed by SDS software (Version 2.4, Applied Biosystems). The threshold cycles (Ct) were calculated and relative gene expression was analyzed after normalizing for *glyceraldehyde 3-phosphate dehydrogenase* (*GAPDH*), the housekeeping gene. Human primers used are listed in Supplementary Table 1.

### Statistical analysis

The *in vitro* data are presented as mean±S.E.M. using the GraphPad Prism version 5.01 (GraphPad for Science, San Diego, CA, USA). Statistical significance was calculated by two-way Student's *t*-test and multiple comparisons between different groups were performed by one-way ANOVA with Bonferroni post-test unless otherwise mentioned in the figure legends. The *P*-values of <0.05 were assumed as statistically significant for all the tests.

## Abbreviations

ALK4: activin-like kinase 4
bFGF: basic fibroblast growth factor
BSA: bovine serum albumin
C/EBP-*β*: CCAAT-enhancer-binding protein-*β*
CLAS: classical
COL5A1: collagen 5A1
EMT: epithelial-to-mesenchymal transition
EGFR: epidermal growth factor receptor
FCS: fetal calf serum
GAPDH: glyceraldehyde 3-phosphate dehydrogenase
GBM: glioblastoma
G-CIMP: CpG island methylator phenotype
GFAP: glial fibrillary acidic protein
H&E: hematoxylin and eosin
HRP: horseradish peroxidase
*IDH1*: *isocitrate dehydrogenase 1* gene
IHC: immunohistochemistry
MES: mesenchymal
MGMT: methylguanine methyltransferase
MMP9: matrix metallopeptidase 9
MRI: magnetic resonance imaging
MTS: (3-(4,5-dimethylthiazol-2-yl)-5-(3-carboxymethoxyphenyl)-2-(4-sulfophenyl)-2H-tetrazolium)
N: neural
NF1: neurofibromatosis type-1
NF-*κ*B: nuclear factor *κ*-light-chain-enhancer of activated B cells
OCT-4: octamer-binding transcription factor 4
OLIG2: oligodendrocyte transcription factor
PDGFR*α*: platelet-derived growth factor receptor-*α*
PN: proneural
qRT-PCR: quantitative real-time PCR
SDS-PAGE: sodium dodecyl sulfate-polyacrylamide gel electrophoresis
siRNA: small interfering RNA
SOX2: sex determining region Y-box 2
STAT3: signal transducer and activator of transcription 3
TAZ: transcriptional coactivator with PDZ-binding motif
TNF*α*: tumor necrosis factor-*α*
TGF-*β*: transforming growth factor-*β*
WT: wild type
ZEB1: zinc-finger E-box-binding homeobox 1
