# Supplementary figures and images for: TGF-β is an inducer of ZEB1-dependent mesenchymal transdifferentiation in glioblastoma that is associated with tumor invasion
Source: Cell Death Dis. 2014 Oct 2;5(10):e1443–. doi: 10.1038/cddis.2014.395 (PMC4649508; doi:10.1038/cddis.2014.395)

Supplementary Figure 1.

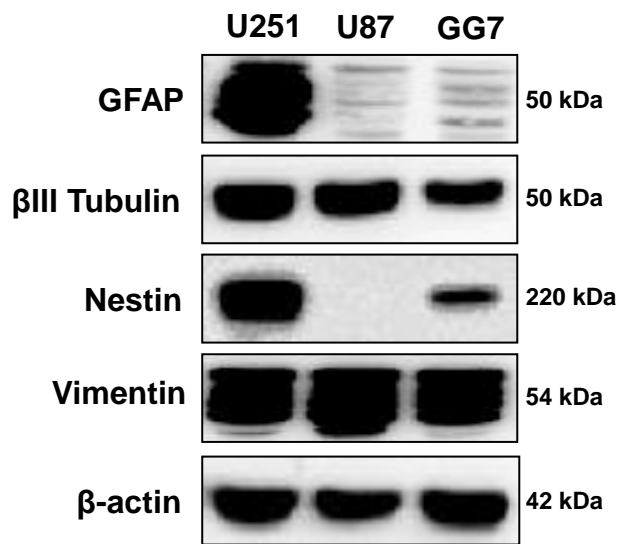

Supplement: Supplementary Figure 1 [file cddis2014395x1.pdf]

Supplementary Figure 2.

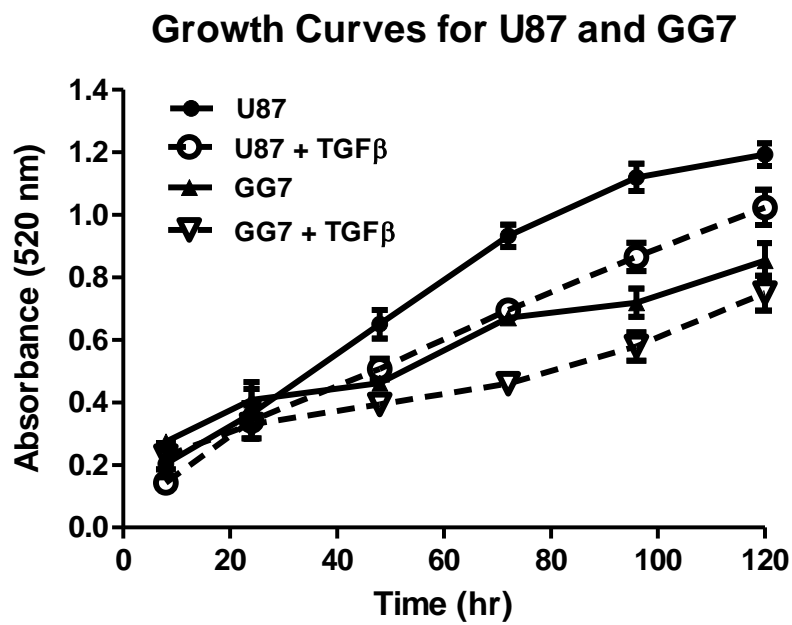

Supplement: Supplementary Figure 2 [file cddis2014395x2.pdf]

Supplementary Figure 3.

a

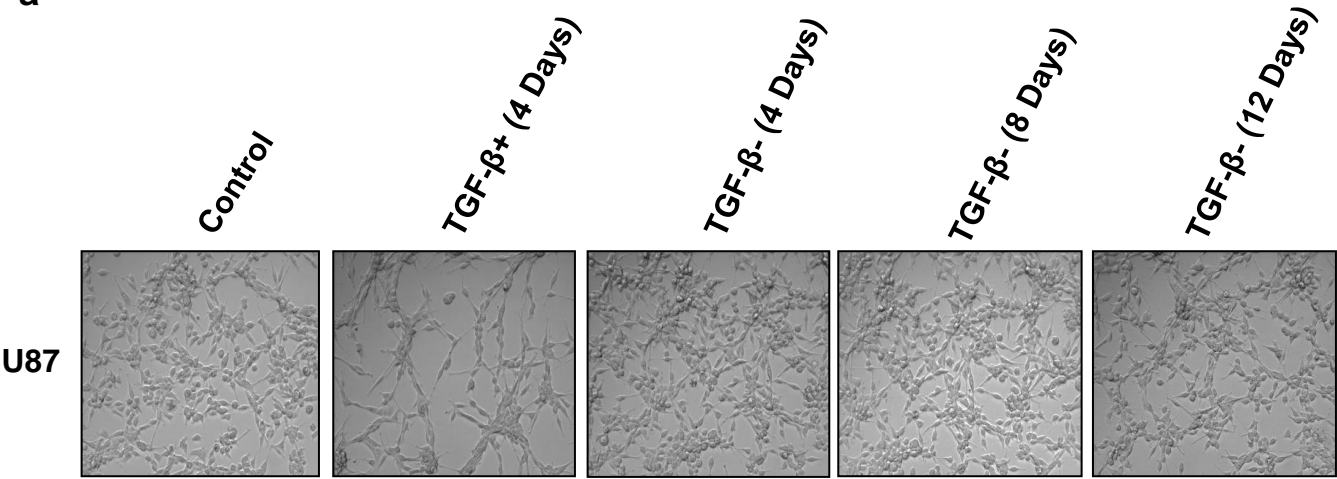

b

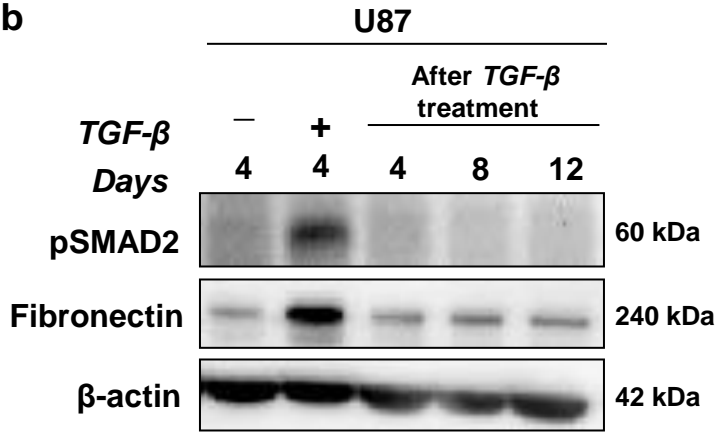

Supplement: Supplementary Figure 3 [file cddis2014395x3.pdf]

Supplementary Figure 4.

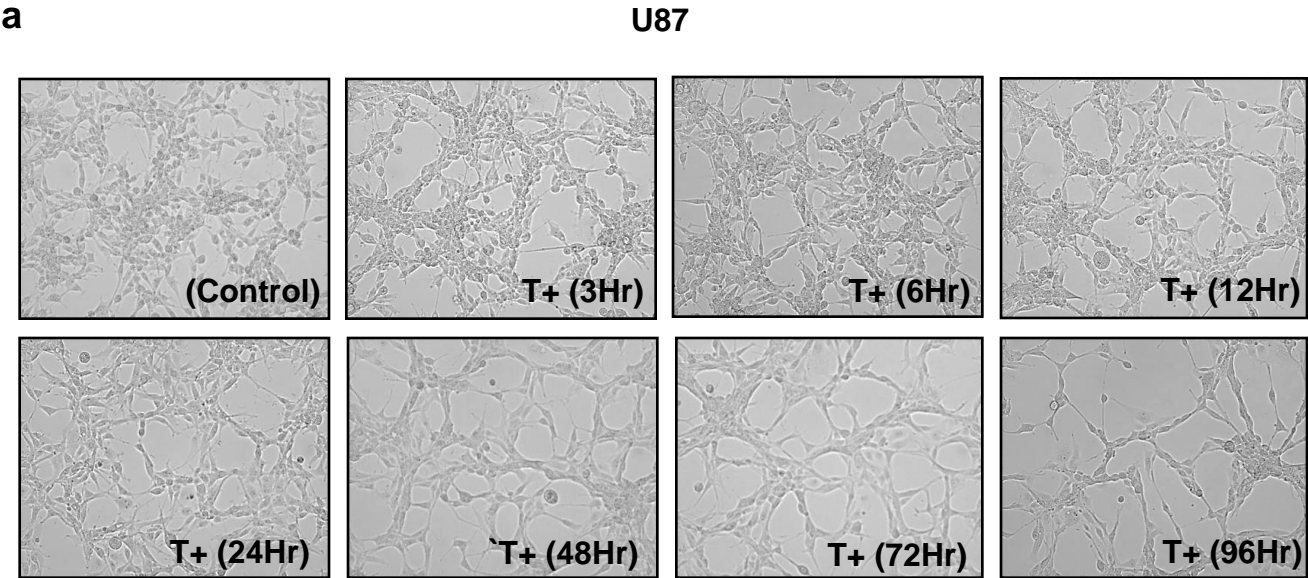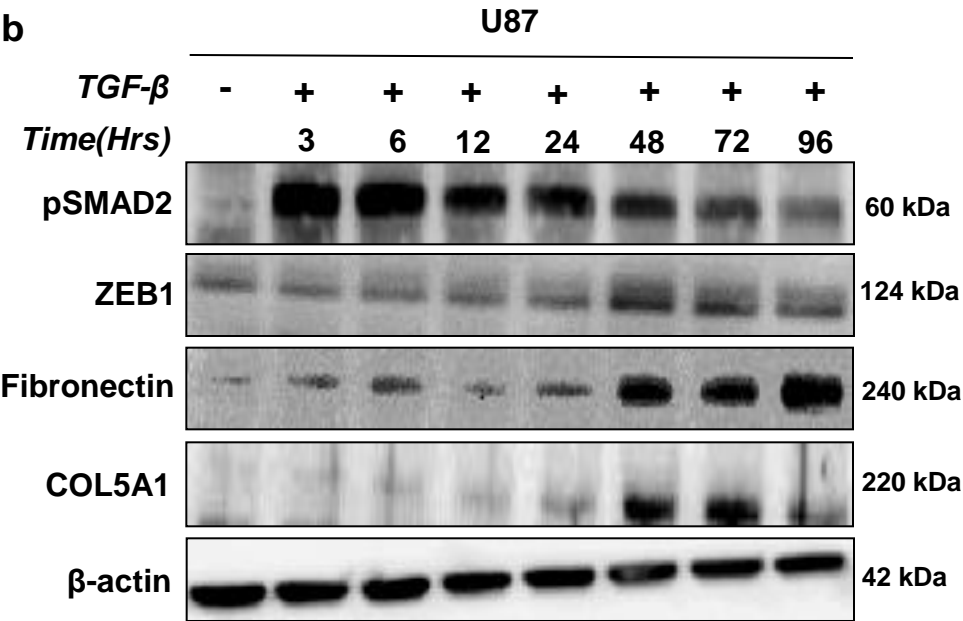

Supplement: Supplementary Figure 4 [file cddis2014395x4.pdf]

Supplementary Figure 5.

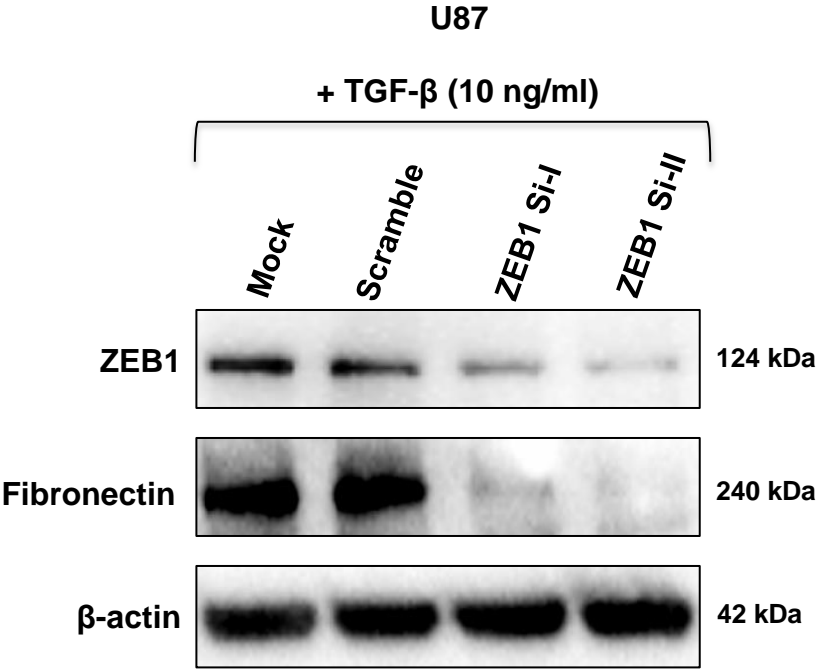

Supplement: Supplementary Figure 5 [file cddis2014395x5.pdf]

Supplementary Figure 6.

a

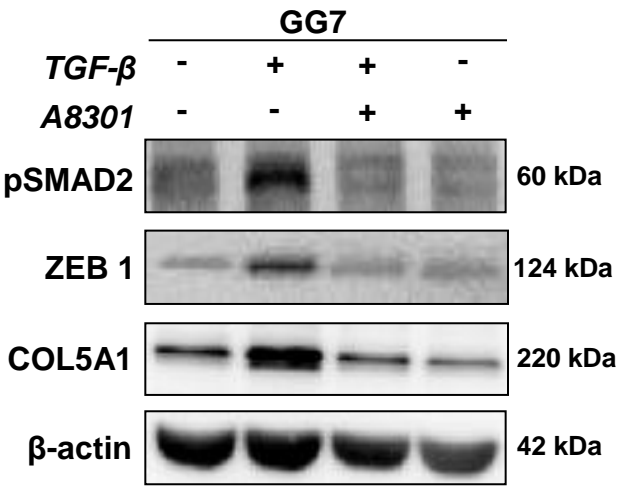

b

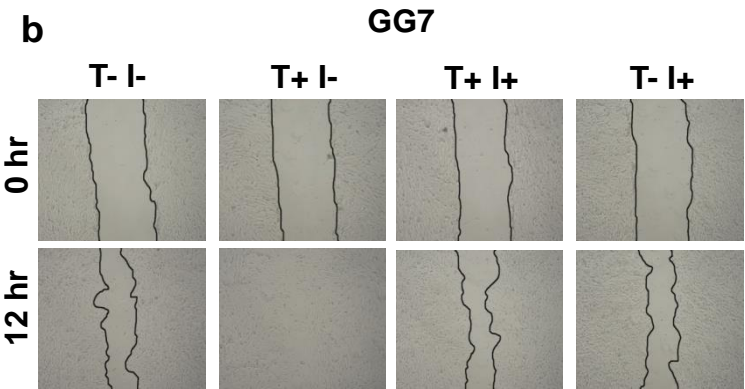

c

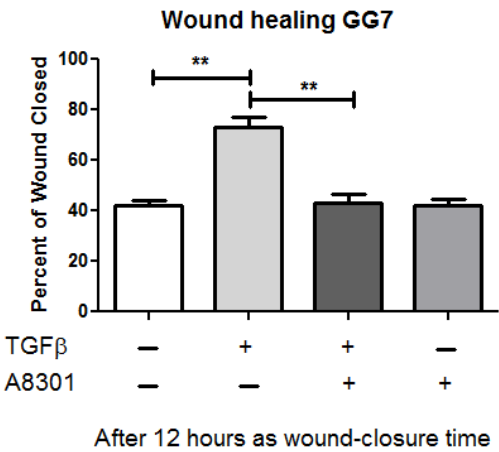

Supplement: Supplementary Figure 6 [file cddis2014395x6.pdf]

Supplementary Figure 7.

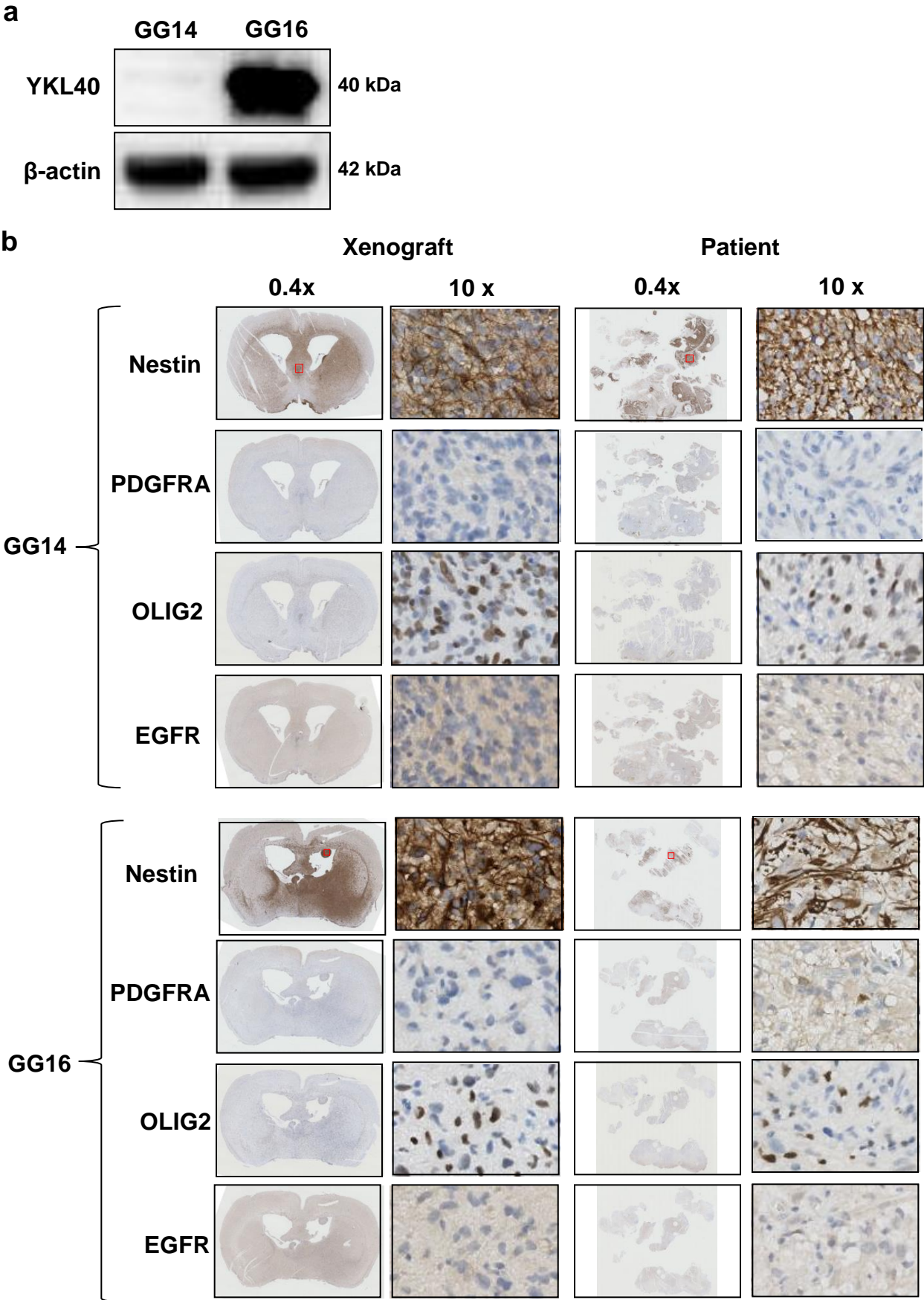

Supplement: Supplementary Figure 7 [file cddis2014395x7.pdf]

Supplementary Figure 8.

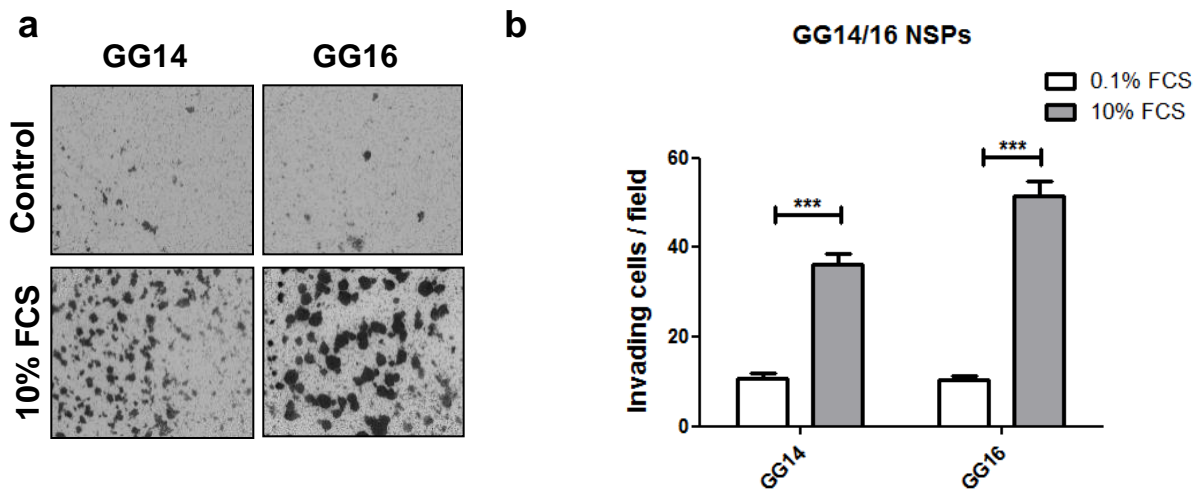

Supplement: Supplementary Figure 8 [file cddis2014395x8.pdf]
