# Supplementary material for: TGF-β is an inducer of ZEB1-dependent mesenchymal transdifferentiation in glioblastoma that is associated with tumor invasion
Source: Cell Death Dis. 2014 Oct 2;5(10):e1443–. doi: 10.1038/cddis.2014.395 (PMC4649508; doi:10.1038/cddis.2014.395)
Supplement: Supplementary Information [file cddis2014395x9.doc]

**Supplementary Figure 1**. Western blots comparing the expression levels of β3 tubulin, GFAP, Nestin and Vimentin in U251, U87 and GG7 cell lines.

**Supplementary Figure 2**. MTS assay showing the proliferative rates of U87 and GG7 cells in the presence and absence of TGF-β.

**Supplementary Figure 3.** Mesenchymal phenotype following withdrawal of TGF-β in U87 cells. **(a)** U87 cells were exposed to TGF-β (10 ng/ml) for 4 days leading to a mesenchymal morphological change. Upon withdrawal of TGF-β the U87 cells reverted back to their original morphology at the day 4 in the absence of TGF-β and continued to maintain their original morphology when followed for 3 passages (12 days). **(b)** Western blots showing loss of pSMAD2 and Fibronectin expression after TGF-β withdrawal.

**Supplementary Figure 4.** Time course experiment of TGF-β-induced changes in morphology and protein expression **(a)** U87 cells where exposed to TGF-β (10ng/ml) for different time points starting from 3 hrs up to 96 hrs. Morphological changes are getting apparent at 24 hrs and as time progress this phenotype gets even more pronounced. **(b)** Western blots showing concurrent appearance of Fibronectin and COL5A1 expression together with ZEB1 induction.

**Supplementary Figure 5.** Western blot demonstrating the downregulation of ZEB1 and mesenchymal marker Fibronection following ZEB1 siRNA transfection and 24 hrs later exposure of the cells to TGF-β (10ng/ml) for 48 hrs.

**Supplementary Figure 6**: GG7 cells acquire further enhanced mesenchymal properties and migration upon exposure to TGF-β (a) Exposure of mesenchymal GG7 cells to TGF-β show pSMAD2 induction along with upregulation of ZEB1 and mesenchymal marker COL5A1. (b) Representative wound healing assays indicating enhanced migratory potential in GG7 cells following TGF-β administration; quantification (n=3, **p<0.01) is shown in (c).

**Supplementary Figure 7.** GG14 and GG16 cell lines display differential expression of YKL40 but the corresponding xenografts showed similar patterns of marker expression as the parental patient tumor. (a) Western blot showing differential expression of YKL40 in GG14 and GG16 neurospheres (b) Immunohistochemical staining comparing expression of the indicated proteins in GG14 and GG16 xenografts to the corresponding patient material. Nestin-positive tumor cells show considerable similarity in the expression of PDGFR-α, OLIG2 and EGFR.

**Supplementary Figure 8.** Transwell assays comparing the invasive capacity of GG14 and GG16 cells. Representative membranes are shown **(a)** and quantification (n=3, ***p<0.001) **(b)**.

**Supplementary Table 1**. Sequences mRNA primers

| **mRNA Primer** | **Sequence** |
| --- | --- |
| ***GAPDH* Frw** | 5’ CACCACCATGGAGAAGGCTGG 3` |
| ***GAPDH* Rev** | 5` CCAAAGTTGTCATGGATGACC 3` |
| ***ZEB1* Frw** | 5` GCACCTGAAGAGGACCAGAG 3` |
| ***ZEB1* Rev** | 5` TGCATCTGGTGTTCCATTTT 3` |
| ***Col5A1* Frw** | 5` GCATTTCCCGAGGACTTCTCC 3` |
| ***Col5A1* Rev** | 5` AATCTGCTGGATACCCTGCTC 3` |
| ***Fibronectin* Frw** | 5` TGGACCAAGTTGATGACACC 3` |
| ***Fibronectin* Rev** | 5` CACCAGGTTGCAAGTCAC 3` |
